# Case report: a thiazide diuretic to treat polyuria induced by tolvaptan

**DOI:** 10.1186/s12882-018-0957-7

**Published:** 2018-07-03

**Authors:** Bart J. Kramers, Maatje D. A. van Gastel, Esther Meijer, Ron T. Gansevoort

**Affiliations:** Department of Internal Medicine, Division of Nephrology, University Medical Center Groningen, University of Groningen, PO Box 30.001, 9700 RB Groningen, The Netherlands

**Keywords:** ADPKD, Tolvaptan, Hydrochlorothiazide, Polyuria, Polycystic kidney disease

## Abstract

**Background:**

Currently, the vasopressin V2 receptor antagonist tolvaptan is the only available treatment for autosomal dominant polycystic kidney disease (ADPKD), but there are tolerability issues due to aquaretic side-effects such as polyuria. A possible strategy to ameliorate these side-effects may be addition of a thiazide diuretic, this is an established treatment in nephrogenic diabetes insipidus, a condition where vasopressin V2 receptor function is absent.

**Case presentation:**

We describe a 46-year-old male ADPKD-patient, who was prescribed tolvaptan, which caused polyuria of around 5 l per day. Hydrochlorothiazide was added to treat hypertension, which resulted in a marked decrease in urine production. While using tolvaptan, rate of eGFR decline was − 1.35 mL/min/1.73m^2^ per year, whereas after hydrochlorothiazide was initiated this was − 3.97 mL/minute/1.73m^2^ per year.

**Conclusions:**

This case report indicates that while addition of hydrochlorothiazide may improve tolerability of vasopressin V2 receptor antagonists, co-prescription should only be used with great scrutiny as it may decrease tolvaptan effect on rate of ADPKD disease progression.

## Background

Autosomal dominant polycystic kidney disease (ADPKD) is characterized by the formation of numerous cysts in both kidneys, leading to renal function loss, with eventually need for renal replacement therapy in the majority of patients [1]. ADPKD is the most common hereditary renal disease and occurs in 3 to 4 per 10,000 people in the general population [2], accounting for 10% of all patients receiving renal replacement therapy [3].

Tolvaptan, a vasopressin V2 receptor antagonist, has recently been registered as the first treatment for ADPKD in Europe, Japan and Canada. In the TEMPO 3:4 trial, performed in ADPKD patients with relatively preserved kidney function and large kidneys, tolvaptan slowed the annual rate of renal function decline by 26% from − 3.70 to − 2.72 mL/min/1.73m^2^ when compared to placebo [4]. Recently, the REPRISE trial demonstrated tolvaptan efficacy in later-stage ADPKD [5]. Being a vasopressin V2 receptor antagonist, tolvaptan causes aquaretic side-effects, such as polyuria (6.0 ± 1.8 l per day), thirst, nocturia and polydipsia [4, 6]. These side-effects impact quality of life and were the main reason for tolvaptan discontinuation in the major trials.

Additional treatment to reduce polyuria may be an option to improve tolvaptan tolerability. Since the polyuria is caused by a pharmacological blockade of the vasopressin V2 receptor, it is of interest to study drugs that are known to effectively reduce polyuria in a situation when vasopressin V2 receptor function is absent, such as in patients with nephrogenic diabetes insipidus. In such patients hydrochlorothiazide (HCT) is known to reduce polyuria by up to 50% [7–9].

The Summary of Medicinal Products Characteristics of tolvaptan cautions that HCT is relatively contraindicated during tolvaptan use, because of the theoretical risk that concomitant use of tolvaptan (an aquaretic) and HCT (a diuretic, or better a saluretic), may cause severe dehydration [10].

In our center, 33 patients started tolvaptan treatment as part of the TEMPO 3:4 trial [4]. We report data on one of these patients, who received HCT despite the aforementioned relative contraindication, as treatment for difficult-to-manage hypertension. This thiazide diuretic influenced his urinary volume notably.

## Case presentation

A 46-year-old Caucasian male was assigned to tolvaptan treatment as part of the TEMPO 3:4 trial in 2008. ADPKD had been diagnosed by ultrasound in 1998, which was performed because of hypertension and a positive family history for ADPKD. DNA analysis later showed a *PKD2* mutation. In 2008, serum creatinine level was 94 μmol/L, with an eGFR of 83 mL/min/1.73m^2^ as calculated by the CKD-EPI formula [11]. Total kidney volume (TKV) was 2351 mL, and height adjusted TKV 1292 mL/m, corresponding to MAYO risk class 1D [12]. Urine showed microalbuminuria (albumin: creatinine ratio 4.75 g/mmol). Pre-tolvaptan, his 24-h urine volume was 1300 mL.

In that same year, tolvaptan was initiated and uptitrated to the maximum dose of 120 mg per day (90/30 mg) within three weeks. After completion of the TEMPO 3:4 trial, tolvaptan was stopped for one month. Thereafter tolvaptan was re-started as part of a compassionate use program. In 2015 this patient developed hypertension despite use of an angiotensin II receptor blocker (losartan 100 mg q.d.) and a beta-blocker (metoprolol 100 mg b.i.d.). Previously an alpha-blocker and a calcium antagonist had led to intolerable side-effects. Therefore HCT was started at a dose of 12.5 mg q.d. that was well-tolerated, and was later increased to 25 mg q.d.

**Fig. 1:**
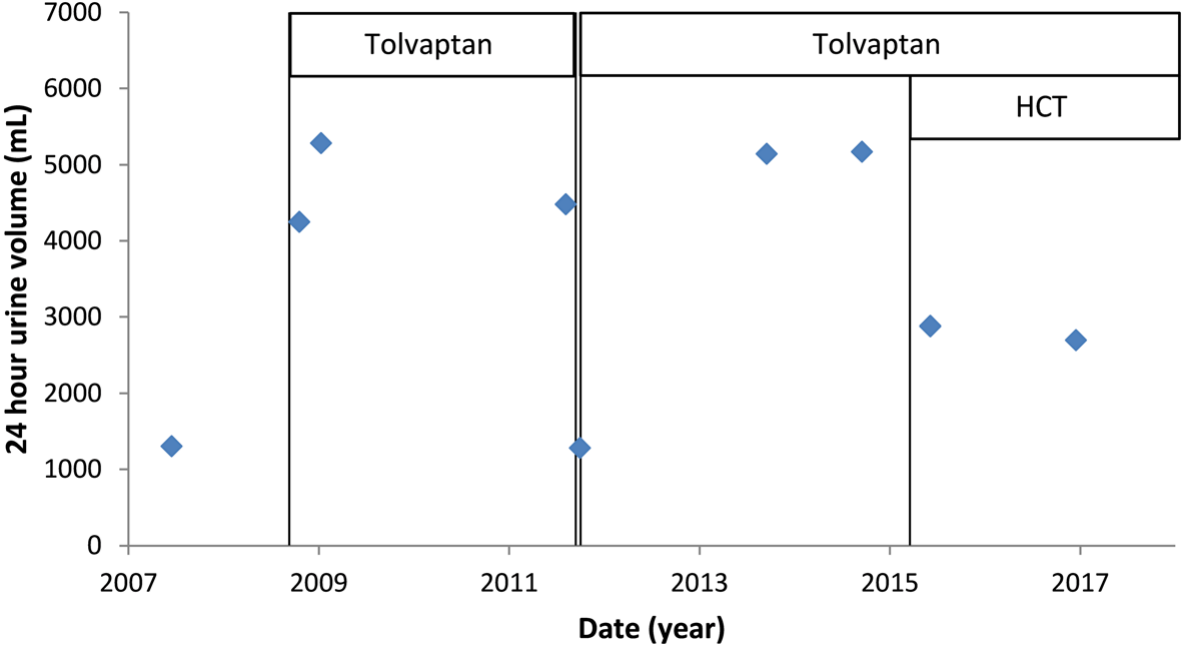
24-h urine volume over time. Urine production increased notably after initiation (in 2008) and re-initiation (in 2011) of tolvaptan. After hydrochlorothiazide (HCT) was added to tolvaptan treatment in 2015, urine volume decreased by 43%

Urinary volumes before and during tolvaptan treatment are shown in Fig. 1. In 2011, this patient collected 24-h urine once during the month in which tolvaptan was temporarily stopped. At that time urine volume was 1280 mL and urine osmolality 632 mOsm/kg. Mean 24-h urine volume (based on five measurements) on tolvaptan before HCT initiation was 4867 mL, mean urine osmolality was 212 mOsm/kg (range 164–250 mOsm/kg). After initiation of HCT (12.5 mg q.d.) 24-h urine production declined to 2878 mL, while urine osmolality increased to 290 mOsm/kg. After the increase of HCT to 25 mg q.d. the last 24-h urine collection measured 2699 mL and 280 mOsm/kg. Mean 24-h urine volume had declined during HCT co-treatment by 2078 mL (43%).

24-h creatinine excretion was used to verify whether urine collections were complete, assuming unchanged muscle mass over time creatinine excretion should be similar between urine collections. Mean 24-h creatinine excretion before start of HCT was 18.1 mmol/24 h, and after start of HCT 16.7 mmol/24 h, indicating no difference in urine collected.

During the nine-year treatment period, serum electrolytes were measured 37 times and stayed within the normal range during the whole period. These electrolytes include potassium (range 3.9–4.6 mmol/L), sodium (range 136–144 mmol/L) and calcium (range 2.34–2.57 mmol/L). There were no differences in average electrolyte concentration between the period with tolvaptan monotherapy and the period with tolvaptan-HCT combination therapy.

**Fig. 2:**
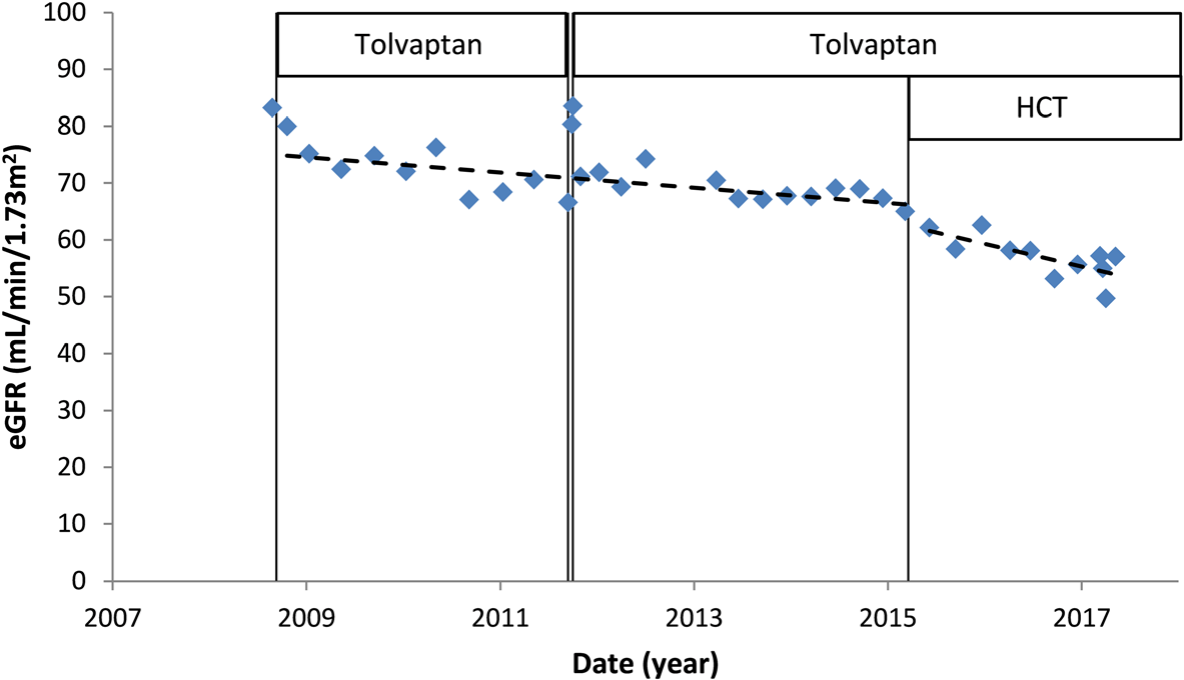
eGFR over time. Tolvaptan has a hemodynamic effect on eGFR (as calculated by the CKD-EPI formula) that occurs acutely after start of the drug, that is reversible after stopping (as can be seen in 2011). After hydrochlorothiazide (HCT) was started in 2015 the rate of eGFR decline seems steeper. Separate trend lines are shown for eGFR decline on tolvaptan monotherapy and for eGFR decline on tolvaptan-HCT combination therapy

eGFR declined from 83 mL/min/1.73m^2^ in 2008 to 57 mL/min/1.73m^2^ in 2017 (Fig. 2). While on tolvaptan monotherapy the slope of eGFR decline was − 1.35 mL/min/1.73m^2^ per year, whereas this was − 3.97 mL/minute/1.73m^2^ per year while on tolvaptan-HCT combination therapy.

Copeptin, a surrogate marker of vasopressin [13], was measured a total of five times, all in a fasting state around the same time in the morning. In the three years on tolvaptan monotherapy it was measured twice, with values of 24.4 and 20.5 pmol/L. In the month the patient temporarily stopped using tolvaptan, copeptin dropped to 11.1 pmol/L. Thereafter, tolvaptan was reinitiated and copeptin increased to 23.3 pmol/L. Copeptin was highest during tolvaptan-HCT combination therapy: 29.7 pmol/L.

## Discussion

HCT has been an established treatment for polyuria in nephrogenic diabetes insipidus for over 50 years [7] and is known to lower urine output by up to 50% within 2–4 days [7–9]. The mechanism explaining this paradoxical antidiuretic effect has never been fully elucidated. Historically, the most widely accepted theory is that HCT blocks the NaCl-cotransporter in the distal convoluted tubule, leading to diminished sodium reabsorption, and as a result extracellular volume contraction, as well as an acute decrease in GFR [14, 15]. Consequently, more sodium and water are reabsorbed in the proximal tubule, less fluid is delivered to the collecting duct, and total urine volume is decreased.

When HCT was started during tolvaptan use, no side-effects were reported by our patient, nor were there any electrolyte abnormalities or signs of dehydration noted. Some clues as to what possible side-effects of tolvaptan-HCT combination treatment may be on rate of ADPKD progression can be derived from the copeptin levels that we measured as surrogate for plasma vasopressin levels [13]. An increase in level of agonist (vasopressin), while remaining on the same dose of antagonist (tolvaptan), could hypothetically attenuate the effect of tolvaptan in slowing eGFR decline. In our patient, copeptin level rose from 22.7 on tolvaptan monotherapy, to 29.7 pmol/L (single measurement) on tolvaptan-HCT therapy, suggesting that HCT caused an increase in plasma copeptin. An increase in copeptin is in accordance with the traditional view that HCT causes a decrease in extracellular volume, which leads to a rise in vasopressin. The evidence for this view however, is limited. The scarce available data that show increased vasopressin activity in patients using HCT was collected in severely hyponatremic patients who received high doses of HCT (mostly ≥100 mg) [16, 17]. In contrast, one could argue that in patients with lower doses of HCT and less hypovolemia, vasopressin could also be suppressed in response to decreased plasma osmolality caused by increased saluresis [18].

Increased copeptin levels could be unfavorable, as upregulation of the vasopressin pathway in ADPKD is associated with more rapid disease progression [19]. In our one patient, there is indeed the suggestion of an acceleration in the rate of eGFR decline after initiation of HCT, from − 1.35 mL/min/1.73m^2^ per year to − 3.97 mL/minute/1.73m^2^ per year (Fig. 2). This finding of a possible increased rate of eGFR decline after HCT initiation should be interpreted with caution, as evidence for the possible deleterious effects of (thiazide) diuretics on ADPKD disease progression is largely lacking. There is one study that compared treatment with ACE-inhibitors and diuretics in hypertensive ADPKD-patients [20]. In this small, non-randomized trial, treatment with diuretics was associated with significantly steeper rate of decline in creatinine clearance during 5 years of follow-up, that may point to a deleterious effects of diuretics.

Evidence regarding the combination of HCT with tolvaptan is equally scarce. In the TEMPO trials and the REPRISE trial, concomitant diuretic use was an exclusion criterion [4, 5, 21]. To our knowledge, only one study specifically investigated the effects of concomitant HCT and tolvaptan use. In that study co-administration of a single dose of 30 mg tolvaptan and 100 mg HCT was compared to administration of a single dose of 30 mg tolvaptan alone in twelve healthy subjects [22]. No significant effect of adding HCT to tolvaptan treatment on vasopressin concentration was noted. In contrast to our findings, polyuria was also not affected by concomitant HCT and tolvaptan use. However, to fully disclose the potential antidiuretic effect of HCT, volume contraction and therefore multiple dosing will be necessary. The effects of low-dose HCT concomitant to tolvaptan on vasopressin concentration require additional research before firm conclusions can be drawn.

A strength of this report is that at the moment HCT was initiated neither the patient, nor the physician were aware of possible effects of HCT usage on urine production, limiting the risk of bias. Urine volumes in this patient were only checked retrospectively.

## Conclusion

Our case suggests that prescription of HCT during tolvaptan use in ADPKD may lead to less polyuria. This improvement in tolerability, however, was accompanied by a rise in copeptin and a possibly accelerated rate of eGFR decline. Furthermore, there theoretically may be risks with respect to hydration status and serum electrolyte concentrations during combined tolvaptan-HCT use, although we found no indications for this in our patient. Taken together these findings indicate that, until further research suggests otherwise, tolvaptan-HCT co-prescription should only be used with great scrutiny.

## Abbreviations

ADPKD: Autosomal dominant polycystic kidney disease
eGFR: Estimated glomerular filtration rate
HCT: Hydrochlorothiazide
TKV: Total kidney volume
